# Clozapine in Treatment-Resistant Bipolar Disorder With Suicidality. Three Case Reports

**DOI:** 10.3389/fpsyt.2019.00520

**Published:** 2019-07-19

**Authors:** Alina Wilkowska, Mariusz S. Wiglusz, Wiesław J. Cubała

**Affiliations:** Department of Psychiatry, Medical University of Gdańsk, Gdańsk, Poland

**Keywords:** clozapine, bipolar disorder, suicidal ideation, bipolar depression, treatment resistance

## Abstract

Bipolar disorder is associated with a high risk of suicide attempts and suicide deaths. The suicide mortality of people with bipolar disorder is approximately 25 times higher than the general population. No approved pharmacological strategies for suicidal thoughts and attempts in bipolar disorder have been introduced so far, and lithium remains as the first-line treatment for suicidal subjects. Clozapine is also a potentially good candidate for this indication. This case series represents three treatment-resistant bipolar patients with severe suicidal ideation who responded to low-dose clozapine treatment.

## Background

Despite growing evidence for the effectiveness of new pharmacological strategies in bipolar disorder (BD), the number of studies on pharmacotherapy of treatment-resistant cases is scarce. One of the medications used for this indication is clozapine. Although clozapine lacks regulatory approval for use in any phase of BD, it has been shown to be useful in treatment-resistant BD (TRBD), decreasing the number of hospitalizations (1) and is associated with symptomatic and functional improvement (2). Clozapine probably reduces aggressive behavior in young patients with TRBD (3), and it seems to be effective in reducing the number of hospitalizations and emergency room (ER) visits, including the ones due to self-harm and overdose (4). One case report presents its antidepressant effect (5) and another its anti-suicidal effect (6).

Suicidal ideation and behaviors in BD consist a significant clinical problem (7, 8). Suicide accounts for 15% to 20% of deaths among BD patients (9, 10). The ratio of suicidal attempts among BD patients is much lower (∼ −3:1) than that in the general population (∼30:1); however, it has a high lethality (11). Suicidal acts appear mostly in association with severe depressive or mixed states. For today, there are no approved pharmacological interventions for suicidality in BD. Clozapine has been shown to have specific anti-suicidal properties in patients with schizophrenia (12–14). Some authors have suggested that clozapine’s anti-suicidal properties could extend beyond schizophrenia to BD (8, 13).

This case series represents three bipolar patients with severe suicidal ideation who responded to clozapine as an add-on treatment.

### Case 1

A 26-year-old single Caucasian female was admitted to an inpatient psychiatry unit. She was diagnosed with BD manic episodes with psychotic symptoms according to Diagnostic and Statistical Manual of Mental Disorders, 5th Edition. She had a 5-year history of BD with three hospitalizations due to manic episodes, including one involuntary. She also had four episodes of depression and one mixed episode. In the course of her illness, she hardly had any remissions.

On admission, she presented an elevated mood, increased energy and motor activity, loud and aggressive behavior, flights of ideas, tangentiality, delusions of grandiosity, and a decrease in sleep and appetite. She had to be restrained because of self-aggression (banging her head against the wall). She had quit her treatment (olanzapine, lithium, fluoxetine, and lorazepam) a few months earlier without consulting her psychiatrist. Initially, she was treated with haloperidol intramuscularly (up to 20 mg) and olanzapine intramuscularly (up to 20 mg), then zuclopenthixol 50 mg intramuscularly and aripiprazole up to 30 mg with no satisfactory effects. Next, she was treated with valproic acid intravenously up to 3,200 mg with a good antimanic effect, but the drug had to be withdrawn due to thrombocytopenia. Moreover, the patient refused to take lithium carbonate due to the “bad taste of the drug” (in Poland, available lithium carbonate tablets are not coated). Subsequently, she was treated with olanzapine 20 mg together with aripiprazole 30 mg that caused a remission of the manic symptoms, but after about 2 weeks, the patient’s mood had worsened; she became extremely irritable and revealed suicidal thoughts.

At this time, clozapine 100 mg was introduced, and the dose was increased to 200 mg the next day. We observed the resolution of the suicidal thoughts and mood normalization during the first 2 days of clozapine treatment. Unfortunately, there was no improvement as far as her insight, and after a few days, the patient refused to take any medication and was discharged at her own demand.

### Case 2

A 26-year-old Caucasian female was admitted to the inpatient psychiatry unit due to severe bipolar I depression with comorbid borderline personality disorder and epilepsy. During the course of her illness, she was hospitalized four times due to depressive episodes and had two manic episodes without hospitalization. The current depressive episode lasted 6 months prior to admission. She had a low mood and extreme lack of energy, anhedonia, psychomotor retardation, reduced sleep, and suicidal thoughts. She was treated with valproate at a maximum of 1,300 mg/day for 9 weeks (serum level 70 mg/l), but the drug was withdrawn due to hair loss and tremor and lack of effect. Aripiprazole at a max of 30 mg/day for 6 weeks that also did not cause any improvement was administered, and then, we used quetiapine at a max of 550 mg/day that caused partial mood stabilization and also severe constipation causing withdrawal. Additionally, the patient was taking lamotrigine 400 mg/day and lithium carbonate 1,000 mg/day during the whole hospitalization. During treatment with lithium carbonate, we introduced eight intravenously ketamine doses (0.5 mg/kg) that caused an immediate mood improvement lasting about a week. Next, we used risperidone 4 mg without any clinical effect. We added topiramate at a max of 400 mg hoping for a further mood stabilizing effect and weight reduction (the patient had gained 10 kg during the year before hospitalization, and it was a significant problem for her). Next, we introduced fluoxetine at a max of 60 mg/day plus 5-mg olanzapine for 10 weeks—which was associated with a mild antidepressant effect.

Finally, we used clozapine at a max of 100 mg for 8 weeks that caused gradual mood and energy normalization and the withdrawal of suicidal thoughts. The patient was discharged on lamotrigine 400 mg, lithium carbonate 750 mg, clozapine 100 mg, and topiramate 400 mg. During the whole time of her hospitalization, the patient had psychodynamic psychotherapy twice a week and continued it after discharge.

### Case 3

A 42-year-old Caucasian female was admitted to the inpatient psychiatry unit due to a severe depressive episode in the course of BD. Before admission, she had quarreled with her husband, and under the influence of ethanol, she had tried to commit suicide by cutting her wrist. She had never been hospitalized in a psychiatric unit before. During hospitalization, she was treated with venlafaxine 375 mg, lamotrigine 200 mg, and quetiapine 200 mg with a small improvement of her depressive symptoms but no effect on her suicidal ideations. The patient stayed at the hospital for 10 days, subsequently withdrawing consent for further hospitalization—claiming her mental state had much improved. The next day, she came to our outpatient clinic still presenting active suicidal thoughts and depressive symptoms. She refused to be hospitalized but agreed to take medication at home with the close supervision of her family and frequent ambulatory visits (three times a week). The patient had been diagnosed with BD type I 21 years ago. During her illness, she had three episodes of major depression, two manic episodes, and one mixed episode. The current depressive episode lasted 2 months before admission and was related to a relationship crisis due to her marital relations. She had a low mood and anhedonia, psychomotor retardation, reduced sleep, and active, persistent suicidal thoughts that were the leading cause of her concern at the moment.

She agreed to be treated with clozapine up to 100 mg as an add-on treatment to previous drugs. During the next two weeks, the patient improved significantly, with the most prominent anti-suicidal effect after 10 days of clozapine treatment. Although she was still depressed, she did not express any suicidal thoughts, and this effect was present as long as the clozapine was subscribed. Two months later, due to sedation, she tried to decrease the dose of clozapine to 25 mg/day, but suicidal thoughts returned. Since then (2 years), she has been taking clozapine as an add-on treatment with a good clinical effect.

The previously discussed cases constitute the part of the clozapine registry approved by the independent ethics committee of the Medical University of Gdansk (approval number NKEBN/355/2016). The previously mentioned cases are presented according to guidelines for disguising case material.

## Discussion

This case series presents the anti-suicidal effect of clozapine in three TRBP patients (**Table 1**). Clozapine is an effective anti-suicide agent approved in schizophrenia (12–14). Data on the pharmacotherapy of suicidal thoughts and behaviors in BD are very sparse, but it is suggested that clozapine can be effective in BD patients experiencing suicidal ideation (8). No approved strategies for treating suicidal thoughts and behaviors in BD have been introduced so far. These mechanisms seem to be independent of that which provides psychotic symptom relief. Interestingly, psychotic symptoms do not predict a better response to clozapine in bipolar patients compared with those in schizophrenic patients (15), and the doses required for optimal effect in BD may be less than those used for treatment-resistant schizophrenia (16). This subject demands further study.

**Table 1:** Clinical characteristics of the case series.

No.	Age	Gender	Education	Marital status	Current episode	Course of BD	Con-meds	Smoker	Clozapine dose
1	26 years	F	12 years	single	Severe depression with psychotic symptoms	BD type I manic = depressive	–	Yes	200 mg
2	26 years	F	17 years	single	Severe depression without psychotic symptoms	BD type I manic < depressive	Lamotrigine 400 mg, lithium carbonicum 750 mg, and topiramate 400 mg	No	100 mg
3	42 years	F	17 years	married	Severe depression without psychotic symptoms	BP type I Manic < depressive	Lamotrigine 200 mg, wenlafaxine 375 mg, bupropion 300 mg, and quetiapine XR 200 mg	No	100 mg

Clozapine’s clinical efficacy in TRBD has been previously demonstrated (17). The unique and complex pharmacology of this drug is responsible for its effectiveness in the treatment of resistant patients (18).

According to British guidelines, clozapine is worth considering as a treatment option in cases of resistant BD, including rapid cycling (19). It is also recommended for TRBD in the latest version of The World Federation of Societies of Biological Psychiatry Guidelines for the Biological Treatment of Bipolar Disorders (20). The latest Canadian guidelines for the treatment of BD suggest using clozapine as the third-line treatment for acute mania and as an additional agent for the maintenance treatment of bipolar I, treatment-resistant mania (21).

Despite being the first drug to demonstrate a reduction in suicidal behavior in a large RCT, clozapine is used in only 1.5% of bipolar patients (4, 22), suggesting a substantial underutilization of this valuable and relatively inexpensive drug. This is most probably caused by the side effects of clozapine: hematological, cardiovascular, metabolic, neurological, but also sedation, cognition, and the need for frequent monitoring of white blood cells. The risk-benefit profile in long-term treatment of BD needs to be assessed carefully.

Possible mechanisms of reducing suicidal thoughts and behaviors with clozapine probably involve the simultaneous modulation of dopamine, norepinephrine, and serotonin (13), regulation of the hormone system (pregnenolone, cortisol) (23, 24), and intracellular systems—dependent modulation of N-methyl-D-aspartate receptor expression, brain-derived neurotrophic factor upregulation, and regulation of the arachidonic acid cascade (24, 25). The decrease in impulsivity and aggression connected with elevated plasma noradrenalin levels in patients treated with clozapine may be another mechanism responsible for the anti-aggressive/anti-suicidal activity of clozapine (26). Impulsivity and aggressive behavior increase the risk of suicidal acts, and mood stabilizers seem to reduce these symptoms (27). According to Youssef, pregnenolone alterations may be relevant to the neurobiology of suicide in schizophrenia and BD and may constitute a common path for the anti-suicidal effect for clozapine and lithium (28). There are few interesting studies suggesting that newer atypical antipsychotics like quetiapine and asenapine could also reduce suicidal ideation in BD, and they, just like clozapine, should definitely be studied further (29).

## Limitations

Clozapine serum level measurements were not performed. No psychometric scales were reported. Concomitant medications could interfere with the effect of clozapine. The case series does not reflect the wider population nor a causative relationship, and thus, replication in a proof of concept study is warranted in a larger population. Longer follow-up might be warranted.

## Conclusions

Despite the limitations, this case series presents the effectiveness of low-dose clozapine in reducing suicidal thoughts in three TRBD patients. The strategy described did not cause any serious adverse events and is relatively safe, especially in the clinical inpatients setting. Considering a lack of approved treatment for suicidal thoughts and behaviors in TRBD, there is a definite need for further studies in this field. A randomized controlled trial of effectiveness and tolerability in TRBD is warranted. Clozapine provides much promise for the treatment of suicidal thoughts and behaviors in this group of patients, and it seems to be effective, safe, and well tolerated.

## Ethics Statement

The studies involving human participants were reviewed and approved by the Independent Ethics Committee of the Medical University of Gdansk (approval number NKEBN/355/2016). The patients/participants provided their written informed consent to participate in this study. Written informed consent was obtained from the individual(s) for the publication of any potentially identifiable images or data included in this article.

## Author Contributions

AW contributed to the manuscript draft, patient management, and research on the topic. MW contributed to the patient management, conceptualized the study, and corrected the manuscript. WC corrected the manuscript and conceptualized the study.

## Conflict of Interest Statement

The authors declare that the research was conducted in the absence of any commercial or financial relationships that could be construed as a potential conflict of interest.
